# An Electrochemical Aptasensor for Accurate and Sensitive Detection of Exosomes Based on Dual-Probe Recognition and Hybridization Chain Reaction

**DOI:** 10.3390/bios15050302

**Published:** 2025-05-09

**Authors:** Haojie Ma, Jie Li, Mengjia Gao, Yan Dong, Yi Luo, Shao Su

**Affiliations:** State Key Laboratory of Flexible Electronics (LoFE) & Jiangsu Key Laboratory of Smart Biomaterials and Theranostic Technology, Institute of Advanced Materials (IAM), Nanjing University of Posts and Telecommunications, 9 Wenyuan Road, Nanjing 210023, China; 1022233718@njupt.edu.cn (H.M.); 1022233717@njupt.edu.cn (J.L.); 1024233507@njupt.edu.cn (M.G.); 1020061605@njupt.edu.cn (Y.D.); iamyluo@njupt.edu.cn (Y.L.)

**Keywords:** exosome, aptasensor, dual-probe recognition, hybridization chain reaction, electrochemical

## Abstract

The accurate and sensitive detection of tumor-derived exosomes holds significant promise for the early diagnosis of cancer. In this study, an electrochemical aptasensor was developed for the high-performance detection of exosomes by integrating dual-probe recognition and hybridization chain reaction (HCR). A dual-probe recognition unit composed of a MUC1 aptamer (MUC1-Apt) probe and cholesterol probe was designed for capturing target exosomes and reducing the interference from free proteins, significantly improving the accuracy of exosome detection. It should be noted that the dual-probe recognition unit was formed in conjunction with the HCR. Moreover, a large number of biotins were also assembled on the HCR product, which were used to capture avidin–horseradish peroxidase (SA-HRP) for signal amplification. The CD63 aptamer (CD63-Apt) was immobilized on the surface of a gold electrode for specifically capturing exosomes to construct a classical sandwiched structure. The loaded SA-HRP can efficiently catalyze the reaction of 3, 3′, 5, 5′ tetramethylbenzidine (TMB) and hydrogen peroxide (H_2_O_2_) to generate a large electrochemical signal. According to this phenomenon, a linear relationship of this proposed aptasensor was achieved between the electrochemical response and 1 × 10^2^–1 × 10^7^ particles/mL exosomes, with a detection limit of 45 particles/mL. Moreover, the aptasensor exhibited accepted stability and potential clinical applicability. All results proved that this aptasensor has a promising application in exosome-based disease diagnostics.

## 1. Introduction

Breast cancer is the second leading cause of cancer deaths among women worldwide [1,2,3]. Early diagnosis and treatment of breast cancer plays a crucial role in alleviating patients’ pain and prolonging their lives [4,5,6]. Compared with classical biomarkers of cancer antigen (CA) 15-3 [7], carcinoembryonic antigen (CEA) [8], HER2 protein [9], microRNA-21 [10], microRNA-195 [11], and microRNA-125b [12], exosomes have emerged as a promising non-invasive biomarker for the early diagnosis and treatment of breast cancer [13]. An exosome is a type of extracellular vesicle with a diameter of approximately 30–200 nm, which is released by cells and widely present in body fluids [14,15]. Exosomes are rich in various biomolecules, including proteins, lipids, nucleic acids, and various membrane proteins (e.g., CD63, EpCAM, CD9, CD81, and MUC1), which can accurately reflect the pathological changes of diseases with high sensitivity and specificity [16,17]. Great efforts have been devoted to developing methods for exosome detection based on fluorescence [18], colorimetry [19], electrochemistry [20], surface-enhanced Raman scattering (SERS) [21], surface plasmon resonance (SPR) [22], and photoelectrochemical (PEC) techniques [23]. Compared to antibodies, aptamers have been considered as alternative tools to recognize membrane proteins for identifying and detecting exosomes [24]. For example, Chen et al. developed an electrochemical aptasensor for extremely specific and sensitive exosome detection based on a DNA four-way junction (4-WJ)-triggered dual rolling circle amplification (RCA) signal amplification strategy [25]. Under optimal conditions, the designed aptasensor showed a wide linear range (1 × 10^2^–1 × 10^7^ particles/mL), low detection limit (20 particles/mL), high selectivity, and excellent stability for MCF-7-derived exosome detection. He et al. developed a portable aptasensor for rapid and sensitive analysis of tumor-derived exosomes by using a handheld fluorescence meter [26]. Due to the signal amplification of parallel RCA reactions, the portable aptasensor can detect down to 30 particles/mL tumor cell-derived exosomes. More importantly, the results of this aptasensor for the analysis of 16 clinical samples were highly consistent with CT and pathological findings.

It should be noticed that free proteins and lipoproteins may coexist with exosomes due to the separation and purification process of exosomes. As a result, it is imperative to acknowledge that the free proteins from damaged exosomes or cells may compromise the precision of aptasensors for target exosome detection [27]. Therefore, it is necessary to develop new strategies for effectively reducing the interference of free proteins and improving identification and detection accuracy. As a lipid sterol, cholesterol is a fundamental component of biological membranes. Because cholesterol does not interact with free proteins and only inserts into the membrane of exosomes, it is considered as a promising probe for recognizing target exosomes [28]. For example, Cheng et al. designed a cholesterol-based signal probe to specifically capture and detect target exosomes through inserting it into the membrane of exosomes [29]. Combined with gold nanoparticle-labeled inductively coupled plasma mass spectrometry (ICP-MS) and rolling circle amplification (RCA), the designed biosensor can detect 1.5 particles/μL exosomes with high selectivity. To further improve detection accuracy, construction of a dual-probe recognition unit composed of an aptamer and cholesterol has become a popular method to efficiently reduce the interference of free proteins. According to this concept, Zhao et al. explored a dual-probe recognition system to accurately detect exosomes by using aptamers to recognize CD63 protein on the surface of exosomes and cholesterol probes to recognize the lipid bilayer of exosomes [30]. Due to their design, the analytical performance of the developed aptasensors achieved a high consistency with that from nanoparticle tracking analysis (NTA).

Inspired by above studies, an electrochemical aptasensor was designed for the accurate and sensitive detection of exosomes by integrating a dual-probe recognition system with hybridization chain reaction (HCR) signal amplification. This designed recognition system employs two specially recognized probes with distinct functions: one probe is equipped with a MUC1 aptamer (MUC1-Apt) for specifically recognizing the membranal MUC1 protein of exosomes, while the other probe is labeled with cholesterol to facilitate insertion into the lipid bilayer of exosomes. This design can efficiently eliminate the interference from free proteins. Moreover, the detection signal can be largely amplified by HCR and introduced enzymes. Through the specific reaction of biotin and avidin, many streptavidin-labeled horseradish peroxidases (SA-HRPs) were assembled on the long HCR products, which can efficiently catalyze the TMB-H_2_O_2_ reaction to generate an outstanding electrochemical signal. Based on these mechanisms, this electrochemical aptasensor can qualitatively and quantitatively detect breast cancer-derived exosomes with high selectivity, reproducibility, and stability.

## 2. Materials and Methods

### 2.1. Materials, Reagents and Apparatus

The used materials, reagents, and apparatus are listed in the Supplementary Materials. The sequences of the MUC1-Apt probe, CD63-Apt, HP1, and HP2 are recorded in Table S1.

### 2.2. Preparation of Exosome–HCR Complexes

At first, 2 μL 1 μM MUC1-Apt probe, 2 μL 1 μM cholesterol probe, 2 μL 5 μM biotin-labeled hairpin probe 1 (HP1), and 2 μL 5 μM biotin-labeled hairpin probe 2 (HP2) were mixed in a solution containing 20 mM Tris, 10 mM NaCl, and 5 mM MgCl_2_ with a molar ratio of 1:1:5:5, resulting in a final volume of 20 μL. Subsequently, the mixture was incubated at 37 °C for 20 min to form HCR products. Different concentrations of exosomes (20 μL) were mixed with HCR products and incubated at 37 °C for 1 h. During this process, the MUC1-Apt probe and cholesterol probe in the HCR products were successfully bound to the exosomes, forming exosome–HCR complexes. To verify the successful preparation of the exosome–HCR complexes, the zeta potentials of the exosome and the formed exosome–HCR complexes were characterized.

### 2.3. Fabrication of the Electrochemical Aptasensor

Firstly, 5 μL 1 μM CD63 aptamer (CD63-Apt) was assembled on the cleaned gold electrode surface via the classical gold–sulfur (Au-S) bond for incubating 12 h at room temperature, which was defined as CD63-Apt/Au. After washing and drying, 5 μL 5 mM 6-mercapto-1-hexanol (MCH) was used to block the nonspecific remaining sites of CD63-Apt/Au for 1 h at room temperature, which was defined as MCH/CD63-Apt/Au. Then, 5 μL exosome–HCR complexes was incubated on MCH/CD63-Apt/Au for 1 h at room temperature, which was named as exosome–HCR/MCH/CD63-Apt/Au. Subsequently, 5 μL 10 μg/mL SA-HRP solution containing 1% fetal bovine serum (FBS) was incubated with the exosome–HCR/MCH/CD63-Apt/Au for 15 min at room temperature. Finally, the electrochemical signal was tested by using the designed modified electrode to catalyze the H_2_O_2_-TMB reaction system.

Cyclic voltammetry (CV) was performed from −0.2 to 0.6 V at a scan rate of 50 mV/s. Electrochemical impedance spectroscopy (EIS) measurements were performed in 4 mL 0.5 mM [Fe(CN)_6_]^3-/4^ solution containing 0.1 M KCl. The experimental parameters were as follows: open circuit potential, 0.2 V; potential amplitude, 5 mV; and frequency range, 0.1–10,000 Hz. Amperometric detection (i-t) was tested in 4 mL static H_2_O_2_-TMB solution containing 0.4 mM TMB and 10 mM H_2_O_2_ by fixing the potential at 150 mV. The current at the 100th second was selected as the detection current of this biosensor when the redox reaction reached steady state.

## 3. Results

### 3.1. Design Principle of the Electrochemical Aptasensor

**Scheme 1:**
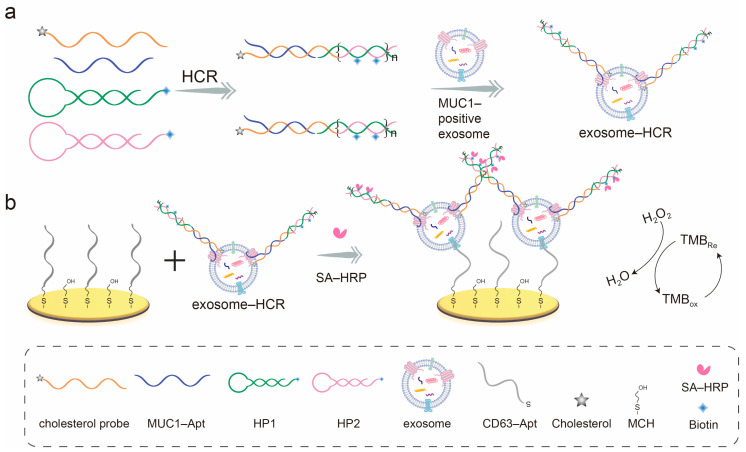
The principle of the electrochemical aptasensor based on a dual-probe recognition system and hybridization chain reaction signal amplification for exosome detection. (**a**) Construction of exosome–HCR complexes, (**b**) Construction of electrochemical aptasensor.

As illustrated in Scheme 1, the electrochemical aptasensor is designed to sensitively and selectively detect breast cancer-derived exosomes. To improve the selectivity, a dual-probe recognition system containing a MUC1-Apt probe and a cholesterol probe was designed for binding to the membranal MUC1 protein on exosomes and inserting into the lipid bilayer of exosomes, respectively (Scheme 1a). As designed, the cholesterol probe hybridized with the MUC1-Apt probe to form a double-stranded DNA structure, which was further used to trigger HCR in the presence of HP1 and HP2. These HCR products were equipped with the exosome recognition system to reduce the interference from free proteins and improve the capture efficiency. In the presence of breast cancer-derived exosomes, HCR products efficiently capture exosomes to form exosome–HCR complexes. For the construction of the aptasensor, CD63-Apt was assembled on the gold electrode for the specific recognition of exosome–HCR complexes. Due to the specific recognition of CD63-Apt and CD63 protein on the surface of exosomes, a classical sandwiched structure was formed on the electrode surface. Subsequently, numerous SA-HRPs bound to the exosome–HCR complexes due to the reaction of avidin–biotin, catalyzing the redox reaction of TMB-H_2_O_2_ to generate an electrochemical signal. According to the change in electrochemical signal, the designed aptasensor can qualitatively and quantitatively detect exosomes with satisfactory results.

### 3.2. Characterization of Exosomes

**Figure 1:**
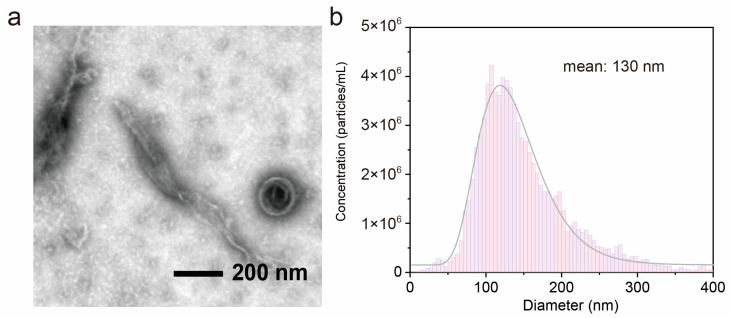
Characterization of exosomes: (**a**) TEM image of the extracted exosomes; (**b**) the size distribution of the extracted exosomes obtained by NTA. The mean diameter of the exosomes is about 130 nm.

Exosomes were extracted from the MCF-7 cell line by differential centrifugation. The morphology and concentration of the extracted exosomes were characterized by a transmission electron microscope (TEM) and nanoparticle tracking analysis (NTA), respectively. As shown in Figure 1a, the extracted exosomes exhibited a characteristic cup-shaped membrane morphology with an average diameter of approximately 130 nm. NTA revealed that the size distribution of the extracted exosomes ranged from 78 to 240 nm, with an average diameter of 130 nm (Figure 1b). These morphological and size characteristics align closely with findings reported in prior studies [31].

### 3.3. Feasibility of the Electrochemical Aptasensor for Exosome Detection

**Figure 2:**
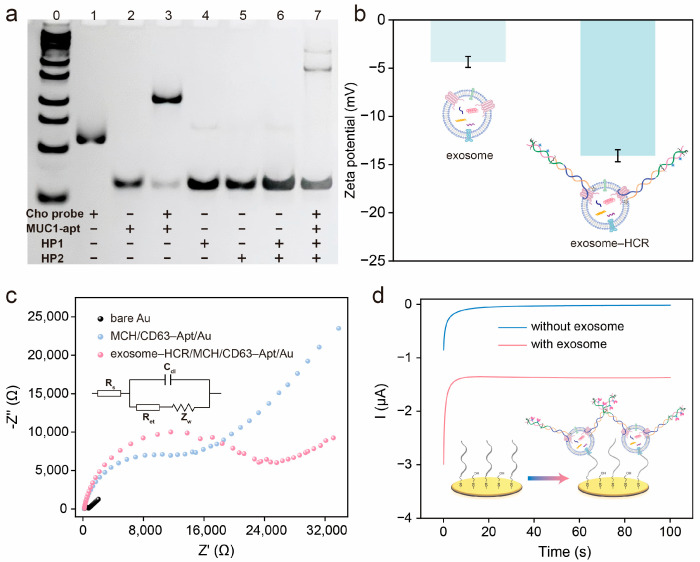
(**a**) Different products were characterized by gel electrophoresis. From lane 0 to lane 7 are DNA markers, the cholesterol probe, MUC1-Apt, the cholesterol probe+MUC1-Apt, HP1, HP2, HP1+HP2, and the cholesterol probe+MUC1-Apt+HP1+HP2, respectively. (**b**) The zeta potentials of exosomes and exosome–HCR complexes, respectively. (**c**) EIS curves of bare Au, MCH/CD63-Apt/Au, and exosome–HCR/MCH/CD63-Apt/Au in 0.5 mM [Fe(CN)_6_]^3-/4-^ solution containing 0.1 M KCl. Inset: an equivalent circuit for the construction of the aptasensor. (**d**) Amperometric responses of this aptasensor for 0 particles/mL and 10^6^ particles/mL exosome detection in buffer.

The feasibility of HCR was firstly evaluated by polyacrylamide gel electrophoresis (PAGE). As shown in Figure 2a, compared to lane 1 and lane 2, a new band was obtained in lane 3 with the hybridization of the cholesterol probe and MUC1-Apt probe. HP1 and HP2 presented a clear band in lane 4 and lane 5, respectively. When HP1 and HP2 coexisted in the solution, only a deeper band was observed in lane 6, suggesting that HP1 and HP2 cannot hybridize with each other. Obviously, new gradient DNA bands with higher molecular weights appeared in lane 7 after mixing the cholesterol probe, MUC1-Apt, HP1, and HP2, indicating the successful formation of HCR products. To evaluate the recognition ability of HCR products towards exosomes, zeta potential was used to characterize the exosomes and exosome–HCR complexes, respectively. As shown in Figure 2b, the zeta potential of the exosomes is −4.35 mV. Following the binding of the HCR products to the exosomes, the zeta potential of exosome–HCR complexes decreases to −14.08 mV. This change suggests that the negatively charged HCR products are effectively assembled onto the exosomes.

Electrochemical impedance spectroscopy (EIS) and cyclic voltammetry (CV) were performed to monitor the fabrication process of this electrochemical aptasensor. As displayed in Figure 2c, the electron transfer resistance (R_et_) of the MCH/CD63-Apt/Au (10601.9 Ω) was larger than that of the bare Au (209.0 Ω), proving the successful immobilization of CD63-Apt on the electrode surface. As designed, the R_et_ of this electrochemical aptasensor significantly increased with the addition of exosome–HCR complexes (21303.6 Ω). The reason was ascribed to the fact that negatively charged exosome–HCR complexes greatly inhibited the electron transfer between the electrode surface and [Fe(CN)_6_]^3-/4-^. The electrochemical behaviors of different preparation processes were also characterized by CV in [Fe(CN)_6_]^4−/3−^solution, which are recorded in Figure S1. The feasibility of this electrochemical aptasensor for exosome detection was verified using the amperometric method. As shown in Figure 2d, a weak current signal was obtained for MCH/CD63-Apt/Au (0.015 μA) in the absence of exosomes. With the addition of exosome–HCR complexes, the electrochemical signal of this aptasensor increased up to 1.79 μA, which is approximately 120 times that of MCH/CD63-Apt/Au. The amplified signal was attributed by the introduction of HCR assembled on the exosome–HCR complexes, which can efficiently catalyze the H_2_O_2_-TMB reaction to generate a large electrochemical signal. These experimental results prove that the designed electrochemical aptasensor can effectively recognize and analyze exosome.

### 3.4. Optimization of Experimental Conditions

**Figure 3:**
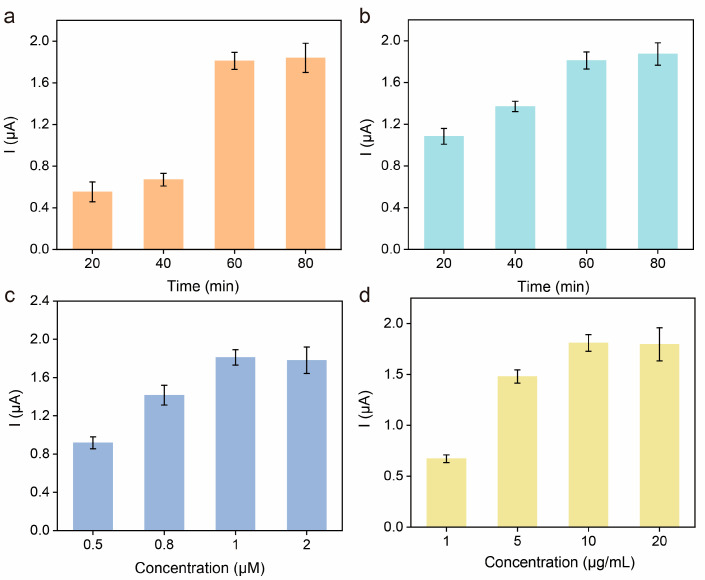
The effect of (**a**) the binding time between HCR products and exosomes, (**b**) the binding time between CD63 aptamer and exosome–HCR complexes, (**c**) the concentration of CD63 aptamer on the electrode surface, and (**d**) the concentration of SA-HRP on the analytical performance of this aptasensor.

To achieve optimal sensing performance, the experimental conditions were optimized, including the binding time between HCR products and exosomes, the incubation time between CD63-Apt and exosome–HCR complexes, the concentration of CD63-Apt immobilized on the electrode surface, and the assembled concentration of SA-HRP. As illustrated in Figure 3a, the electrochemical currents of this aptasensor increased with the increasing binding time between HCR products and exosomes ranging from 20 to 80 min. The electrochemical response reached a plateau when the binding time was 60 min. Therefore, the binding time between HCR products and exosomes was chosen as 60 min. Similarly, 60 min was selected as the optimal binding time between CD63-Apt and exosome–HCR complexes (Figure 3b). Subsequently, the concentration of CD63-Apt immobilized on the electrode was investigated. As shown in Figure 3c, a signal plateau of this aptasensor was obtained when the concentration of CD63-Apt was 1 μM. Therefore, 1 μM CD63-Apt was immobilized on the electrode surface to maximally recognize and capture exosome–HCR complexes. Figure 3d shows that 10 μg/mL SA-HRP was employed to achieve the best analytical performance.

### 3.5. Electrochemical Detection of Exosomes

**Figure 4:**
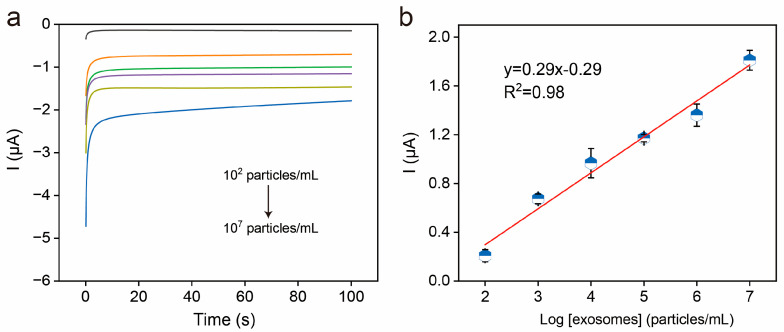
(**a**) Amperometric responses of this aptasensor for 10^2^–10^7^ particles/mL exosome detection. (**b**) The linear relationship between the current values and the logarithm of exosome concentrations.

Under the optimal conditions, the analytical performance of this electrochemical aptasensor was evaluated by varying exosome concentrations. The amperometric responses of the developed aptasensor were tested with increasing exosome concentrations ranging from 10^2^ to 10^7^ particles/mL (Figure 4a). Obviously, more exosomes can cause a larger electrochemical signal. Figure 4b shows that a linear relationship was obtained between the current of this aptasensor and the logarithmic values of 10^2^–10^7^ particles/mL exosomes with an equation of I = 0.29 log [exosome] − 0.29 (R^2^ = 0.98). The limit of detection (LOD) was estimated to be 45 particles/mL (S/N = 3). The detection performance obtained in this work is comparable to or better than several published works (Table 1).

### 3.6. Selectivity, Anti-Interference, Reproducibility, and Stability of the Electrochemical Aptasensor

**Figure 5:**
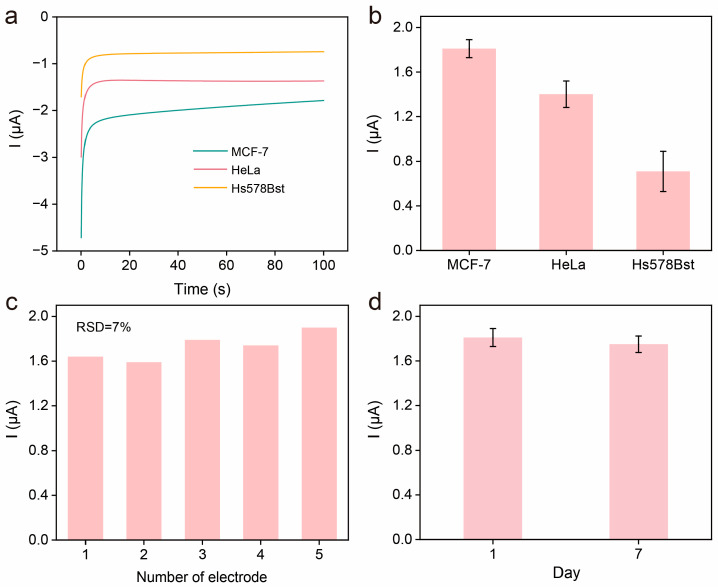
(**a**) Amperometric responses of this aptasensor for exosome detection derived from MCF-7, HeLa, and Hs578Bst, respectively. (**b**) The current values of this aptasensor for exosome detection derived from MCF-7, HeLa, and Hs578Bst, respectively. (**c**) The reproducibility of this aptasensor for exosome detection. (**d**) The storage stability of this aptasensor.

Three kinds of exosomes derived from MCF-7 cells (high MUC1 expression), HeLa cells (moderate MUC1 expression), and Hs578Bst cells (low or negligible MUC1 expression) were used to evaluate the selectivity of this electrochemical aptasensor, respectively. As shown in Figure 5a,b, exosomes derived from MCF-7 cells produced the highest electrochemical signal compared to those derived from HeLa and Hs578Bst cells, suggesting the MUC1 protein expression of MCF-7 cell-derived exosomes was higher than that of HeLa and Hs578Bst cells. It was noted that the current of the aptasensor for MCF-7-derived and HeLa-derived exosome detection was about 1.81 μA and 1.40 μA, respectively. The difference in electrochemical signal represents the different MUC1 expression level of exosomes, which is consistent with previous research findings [37]. These experimental results prove that the developed aptasensor can effectively distinguish the exosomes derived from MCF-7 cells and other tumor cells. In addition, free CD63 protein and free MUC1 protein were selected as potential interfering substances to further validate the anti-interference ability of the aptasensor. As shown in Figure S2, no obvious electrochemical responses were obtained by using this aptasensor for the detection of free CD63 protein and MUC1 protein. Meanwhile, significant current signals were observed by using this aptasensor for 10^4^ particles/mL exosome detection in buffer and 10% fetal bovine serum (FBS) simulated samples, respectively, proving that the dual-probe recognition system significantly enhances the aptasensor’s anti-interference ability.

Then, the reproducibility of the aptasensor was estimated by detecting exosomes at five independent modified electrodes (Figure 5c). The relative standard deviation (RSD) was calculated as 7.0%, suggesting good reproducibility of the aptasensor. Finally, the long-term storage stability was tested by placing the aptasensor in a refrigerator. Figure 5d shows a small decrease in the electrochemical signal after 7 days of storage, proving that this aptasensor has excellent storage stability.

### 3.7. Detection of Exosome in Serum Sample

The standard addition method was employed to evaluate the practical application of this aptasensor. Exosomes were spiked into 10% diluted FBS at 10^2^ particles/mL, 10^4^ particles/mL, and 10^7^ particles/mL, respectively, which were used as simulated samples. According to the results in Table 2, accepted recoveries (97–107%) and RSD (5.27–9.96%) were observed, suggesting that the aptasensor is a promising platform for analyzing exosomes in real samples.

## 4. Conclusions

In this study, an electrochemical aptasensor was successfully developed for high-performance detection of breast cancer-derived exosomes. Taking advantage of the dual-probe recognition system and HCR-amplified strategy, the developed aptasensor can sensitively and selectively detect as low as 45 particles/mL exosomes with high reproducibility and storage stability. As expected, the designed dual-probe recognition system brings excellent anti-interference ability to the aptasensor, which can effectively avoid the influence of free proteins on detection performance. Moreover, the electrochemical aptasensor showed a potential clinical applicability, demonstrating that the proposed aptasensor has broad application prospects in exosome-based disease diagnosis. For further commercial applications, nanozymes can be used instead of HRP to reduce detection costs.

## Figures and Tables

**Table 1:** Comparison of the analytical performance of the developed aptasensor with other reported biosensors for exosome detection.

Methods	Linear Range (Particles/mL)	Limit of Detection (Particles/mL)	Detection Time (Min)	Ref.
Electrochemistry	10^2^−10^7^	20	360	[25]
Fluorescence	10^2^−10^4^	30	328	[26]
Electrochemistry	2.0 × 10^6^−4.0 × 10^8^	1.5 × 10^6^	30	[32]
Electrochemistry	10^5^−4.0 × 10^7^	4 × 10^5^	206	[33]
Fluorescence	1.66 × 10^6^−1.66 × 10^9^	4.8 × 10^5^	250	[34]
Colorimetry	5 × 10^4^−1.0 × 10^6^	5 × 10^4^	150	[35]
Surface-enhanced Raman scattering	5.5 × 10^4^−5.5 × 10^8^	1.7 × 10^4^	60	[36]
Electrochemistry	10^2^−10^7^	45	135	This work

**Table 2:** The performance of this aptasensor for exosome detection in 10% serum.

Sample	Added (Particles/mL)	Detected (Particles/mL)	Recovery (%)	RSD (%)
1	1.00 × 10^2^	1.02 × 10^2^	102.00	9.89
2	1.00 × 10^4^	1.07 × 10^4^	107.00	5.27
3	1.00 × 10^7^	0.97 × 10^7^	97.00	9.96

## Data Availability

Data is contained within the article.

## Supplementary Materials

The following supporting information can be downloaded at https://www.mdpi.com/article/10.3390/bios15050302/s1: Figure S1. CV curves of bare Au, MCH/CD63-Apt/Au, and exosome–HCR/MCH/CD63-Apt/Au in 0.5 mM [Fe(CN)6]3-/4 solution containing 0.1 M KCl.; Figure S2. Anti-interference ability of this aptasensor for the detection of exosomes (*p* ≤ 0.0001); Table S1. DNA sequences required in the experiment (5′ to 3′).

## References

[1] Li Y., Wang B., Lai H., Li S., You Q., Fang Y., Li Q., Liu Y. (2017). Long Non-Coding RNA Crala Is Associated with Poor Response to Chemotherapy in Primary Breast Cancer. Thorac. Cancer.

[2] Dolatkhah R., Dastgiri S., de Bock G.H., Sanaat Z., Ranjkesh M., Jabbaripour P., Pashaee S. (2018). The First Screening Experiences of Breast Cancer in Northwest of Iran. J. Clin. Oncol..

[3] Sebastião A.I., Simões G., Oliveira F., Mateus D., Falcão A., Carrascal M.A., Gomes C., Neves B., Cruz M.T. (2025). Dendritic Cells in Triple-Negative Breast Cancer: From Pathophysiology to Therapeutic Applications. Cancer Treat. Rev..

[4] Sadeghi M., Sadeghi S., Naghib S.M., Garshasbi H.R. (2023). A Comprehensive Review on Electrochemical Nano Biosensors for Precise Detection of Blood-Based Oncomarkers in Breast Cancer. Biosensors.

[5] Mohammadpour-Haratbar A., Boraei S.B., Zare Y., Rhee K.Y., Park S.-J. (2023). Graphene-Based Electrochemical Biosensors for Breast Cancer Detection. Biosensors.

[6] Moroni S., Casettari L., Lamprou D.A. (2022). 3D and 4D Printing in the Fight against Breast Cancer. Biosensors.

[7] Hu X., Cao Y. (2018). CA 15-3 Elevation in U.S. Women without Breast Cancer. J. Clin. Oncol..

[8] Bae S.Y., Lim W., Jeong J., Lee S., Choi J., Park H., Jung Y.S., Jung S.P., Nam S. (2019). The Prognostic Significance of Preoperative Tumour Marker (CEA, CA 15-3) Elevation in Breast Cancer Patients. Ann. Oncol..

[9] Zhou Q., Pan Y., Liang Q., Yang J., Zhu S., Shi H., Li G. (2024). Peptide-Guided Assembly of Silver Nanoparticles for the Diagnosis of HER2-Positive Breast Cancer. Anal. Chem..

[10] Li H., Tie X.J. (2024). Exploring Research Progress in Studying Serum Exosomal miRNA-21 as a Molecular Diagnostic Marker for Breast Cancer. Clin. Transl. Oncol..

[11] Tokumaru Y., Oshi M., Katsuta E., Murthy V., Matsuhashi N., Futamura M., Yoshida K., Takabe K. (2021). Low Expression of microRNA-195 Is a Poor Prognostic Marker for ER-Positive Breast Cancer Patients. J. Clin. Oncol..

[12] Wang Y., Wei Y., Fan X., Zhang P., Wang P., Cheng S., Zhang J. (2020). MicroRNA-125b as a Tumor Suppressor by Targeting MMP11 in Breast Cancer. Thorac. Cancer.

[13] Cao Y., Yu X., Zeng T., Fu Z., Zhao Y., Nie B., Zhao J., Yin Y., Li G. (2022). Molecular Characterization of Exosomes for Subtype-Based Diagnosis of Breast Cancer. J. Am. Chem. Soc..

[14] Kalluri R., LeBleu V.S. (2020). The Biology, Function, and Biomedical Applications of Exosomes. Science.

[15] Shao H., Im H., Castro C.M., Breakefield X., Weissleder R., Lee H. (2018). New Technologies for Analysis of Extracellular Vesicles. Chem. Rev..

[16] Fan Y., Pionneau C., Cocozza F., Boëlle P.-Y., Chardonnet S., Charrin S., Théry C., Zimmermann P., Rubinstein E. (2023). Differential Proteomics Argues against a General Role for CD9, CD81 or CD63 in the Sorting of Proteins into Extracellular Vesicles. J. Extracel. l Vesicles.

[17] Hu X., Cheng S., Luo X., Xian Y., Zhang C. (2023). Polymerase-Driven Logic Signal Amplification for the Detection of Small Extracellular Vesicle Surface Proteins and the Identification of Breast Cancer. Anal. Chem..

[18] Zhou J., Lin Q., Huang Z., Xiong H., Yang B., Chen H., Kong J. (2022). Aptamer-Initiated Catalytic Hairpin Assembly Fluorescence Assay for Universal, Sensitive Exosome Detection. Anal. Chem..

[19] Zhang X., Zhu X., Li Y., Hai X., Bi S. (2023). A Colorimetric and Photothermal Dual-Mode Biosensing Platform Based on Nanozyme-Functionalized Flower-Like DNA Structures for Tumor-Derived Exosome Detection. Talanta.

[20] Wang Y., Jie H., Ye H., Zhang Y., Li N., Zhuang J. (2023). Methylene Blue-Stained Single-Stranded DNA Aptamers as a Highly Efficient Electronic Switch for Quasi-Reagentless Exosomes Detection: An Old Dog with New Tricks. Anal. Chem..

[21] Gong L., Chen B., Tong Y., Luo Y., Zhu D., Chao J., Wang L., Su S. (2024). Two-in-One Sensing Platform for the Detection of Exosomes Based on Molybdenum Disulfide-Based Nanozymes. Sensor. Actuat. B Chem..

[22] Nurrohman D.T., Chiu N.-F., Hsiao Y.-S., Lai Y.-J., Nanda H.S. (2024). Advances in Nanoplasmonic Biosensors: Optimizing Performance for Exosome Detection Applications. Biosensors.

[23] Xia Y., Chen T., Chen W., Chen G., Xu L., Zhang L., Zhang X., Sun W., Lan J., Lin X. (2022). A Dual-Modal Aptasensor Based on a Multifunctional Acridone Derivate for Exosomes Detection. Anal. Chim. Acta.

[24] Zhu C., Li L., Wang Z., Irfan M., Qu F. (2020). Recent Advances of Aptasensors for Exosomes Detection. Biosens. Bioelectron..

[25] Zhao Z., Yang S., Tang X., Feng L., Ding Z., Chen Z., Luo X., Deng R., Sheng J., Xie S. (2024). DNA Four-Way Junction-Driven Dual-Rolling Circle Amplification Sandwich-Type Aptasensor for Ultra-Sensitive and Specific Detection of Tumor-Derived Exosomes. Biosens. Bioelectron..

[26] He Y., Zeng X., Xiong Y., Shen C., Huang K., Chen P. (2024). Portable Aptasensor Based on Parallel Rolling Circle Amplification for Tumor-Derived Exosomes Liquid Biopsy. Adv. Sci..

[27] Abramowicz A., Widlak P., Pietrowska M. (2016). Proteomic Analysis of Exosomal Cargo: The Challenge of High Purity Vesicle Isolation. Mol. Biosyst..

[28] Ercole F., Whittaker M.R., Quinn J.F., Davis T.P. (2015). Cholesterol Modified Self-Assemblies and Their Application to Nanomedicine. Biomacromolecules.

[29] Cheng Y., Xie Q., He M., Chen B., Chen G., Yin X., Kang Q., Xu Y., Hu B. (2022). Sensitive Detection of Exosomes by Gold Nanoparticles Labeling Inductively Coupled Plasma Mass Spectrometry Based on Cholesterol Recognition and Rolling Circle Amplification. Anal. Chim. Acta.

[30] Zhao X., Luo C., Mei Q., Zhang H., Zhang W., Su D., Fu W., Luo Y. (2020). Aptamer-Cholesterol-Mediated Proximity Ligation Assay for Accurate Identification of Exosomes. Anal. Chem..

[31] Wang Y.-T., Cai M.-D., Sun L.-L., Hua R.-N. (2021). A Rapid and Facile Separation–Detection Integrated Strategy for Exosome Profiling Based on Boronic Acid-Directed Coupling Immunoaffinity. Anal. Chem..

[32] Liu D., Tang J., Xu H., Yuan K., Aryee A.A., Zhang C., Meng H., Qu L., Li Z. (2022). Split-Aptamer Mediated Regenerable Temperature-Sensitive Electrochemical Biosensor for the Detection of Tumour Exosomes. Anal. Chim. Acta.

[33] Pallares-Rusiñol A., Moura S.L., Martí M., Pividori M.I. (2023). Electrochemical Genosensing of Overexpressed Gapdh Transcripts in Breast Cancer Exosomes. Anal. Chem..

[34] Pan Y., Wang L., Deng Y., Wang M., Peng Y., Yang J., Li G. (2020). A Simple and Sensitive Method for Exosome Detection Based on Steric Hindrance-Controlled Signal Amplification. Chem. Commun..

[35] Li C., Guo L., Sang X., Jiang X., Wang H., Qin P., Huang L. (2023). Colorimetric Aptasensor Based on Spherical Nucleic Acid-Induced Hybridization Chain Reaction for Sensitive Detection of Exosomes. Talanta.

[36] Pan H., Dong Y., Gong L., Zhai J., Song C., Ge Z., Su Y., Zhu D., Chao J., Su S. (2022). Sensing Gastric Cancer Exosomes with MoS_2_-Based SERS Aptasensor. Biosens. Bioelectron..

[37] Zhang J., Shi J., Liu W., Zhang K., Zhao H., Zhang H., Zhang Z. (2018). A Simple, Specific and “on-off” Type MUC1 Fluorescence Aptasensor Based on Exosomes for Detection of Breast Cancer. Sensor. Actuat. B Chem..

