# Supplementary material for: An Electrochemical Aptasensor for Accurate and Sensitive Detection of Exosomes Based on Dual-Probe Recognition and Hybridization Chain Reaction
Source: Biosensors (Basel). 2025 May 9;15(5):302. doi: 10.3390/bios15050302 (PMC12109912; doi:10.3390/bios15050302)
Supplement: Supplementary file 1 [file biosensors-15-00302-s001.zip › biosensors-3547006-supplementary.pdf]

Supplementary Materials

An electrochemical aptasensor for accurate and sensitive detection of exosomes based on dual-probe recognition and hybridization chain reaction

Haojie Ma, Jie Li, Mengjia Gao, Yan Dong, Yi Luo and Shao Su*

State Key Laboratory of Flexible Electronics (LoFE) & Jiangsu Key Laboratory of Smart Biomaterials and Theranostic Technology, Institute of Advanced Materials (IAM), Nanjing University of Posts and Telecommunications, 9 Wenyuan Road, Nanjing 210023, China; 1022233718@njupt.edu.cn (H.M.); 1022233717@njupt.edu.cn (J.L.); 1024233507@njupt.edu.cn (M.G.); 1020061605@njupt.edu.cn (Y.D.); iamyluo@njupt.edu.cn (Y.L.)

* Correspondence: iamssu@njupt.edu.cn

1. Materials, reagents, and apparatus

The mixed solution of hydrogen peroxide (H_2O_2) and 3, 3′, 5, 5′ tetramethylbenzidine (TMB) was purchased from Neogen (USA). Tris-(hydroxymethyl) aminomethane (Tris), tris(2-carboxyethyl) phosphine hydrochloride (TCEP), and 6-mercapto-1-hexanol (MCH) were received from Sigma-Aldrich (Shanghai, China). PBS (1×), Dulbecco's modified Eagle's medium (DMEM), fetal bovine serum (FBS), and exosome-depleted fetal bovine serum (dFBS) were purchased from Jiangsu Kaiji Biotechnology Co., Ltd (Nanjing, China). All cells used in this work were purchased from American Type Culture Collection (ATCC, USA). Streptavidin–horseradish peroxidase (SA-HRP), bovine serum albumin (BSA), DNA staining dye gel-red, 30% polyacrylamide, tetramethyl ethylenediamine (TEMED), ammonium persulphate (APS), DNA, and RNA were purchased from Sangon Biotech Co., Ltd (Shanghai, China). The DNA and RNA sequences are shown in Table S1. All reagents were analytical grade and used without further purification. The phosphate buffer (PB) was prepared by mixing 0.2 M $Na_2HPO_4 \cdot 12H_2O$ and 0.2 M $NaH_2PO_4 \cdot 2H_2O$. The electrochemical detection solution contained 0.1 M KCl with 0.5 mM $K_3[Fe(CN)_6]$ and $K_4[Fe(CN)_6]$. Aqueous solutions were prepared by using ultrapure water (>18 MΩ cm) obtained from a Millipore water purification system.

All electrochemical measurements were performed on a CHI 660E electrochemical workstation (Shanghai Chenhua Instruments Co., Ltd., China). A gold electrode (2.0 mm in diameter), a platinum wire, and a Ag/AgCl electrode (saturated KCl) were used as the working electrode, the counter electrode, and the reference electrode, respectively. The concentration and particle size analysis data of exosomes were obtained using a nanoparticle tracking analyzer (NTA) (Malvern Instruments Ltd., UK). Transmission electron microscopy (TEM) (Hitachi Ltd.) was used to characterize the morphology of exosomes. Zeta potential was measured using a zeta potential analyzer (Brookhaven Instruments Corporation, USA). Gel imaging experiments were conducted using a Bio-Rad gel imaging system (Bio-Rad Laboratories, USA).

2. Cell culture and exosome extraction

In this study, exosomes derived from the human breast cancer cell line MCF-7 were chosen as the detection model. MCF-7 cells were cultured in DMEM supplemented with 10% FBS and 1% penicillin/streptomycin at 37°C in a 5% CO_2 atmosphere. To extract extracellular vehicles (EVs) from the culture medium, cells were separated once they reached 70−80% confluence. Following two washes with

PBS, the cells were further cultured in serum-free medium for 24–48 h. Subsequently, the supernatant from the serum-free medium was sequentially centrifuged at 200 g for 20 min, 3000 g for 15 min, and 12000 g for 30 min, followed by filtration through a 0.22 μm filter. The final exosome-containing product was purified using a 100 KD ultrafiltration centrifuge tube at 4000 g for 10 min. The isolated exosomes were then resuspended in PBS and stored at −80°C. The morphology of the exosomes was examined using transmission electron microscopy (TEM), and their concentration was determined via nanoparticle tracking analysis (NTA). These exosomes, with precisely measured concentrations, were subsequently utilized as standards.

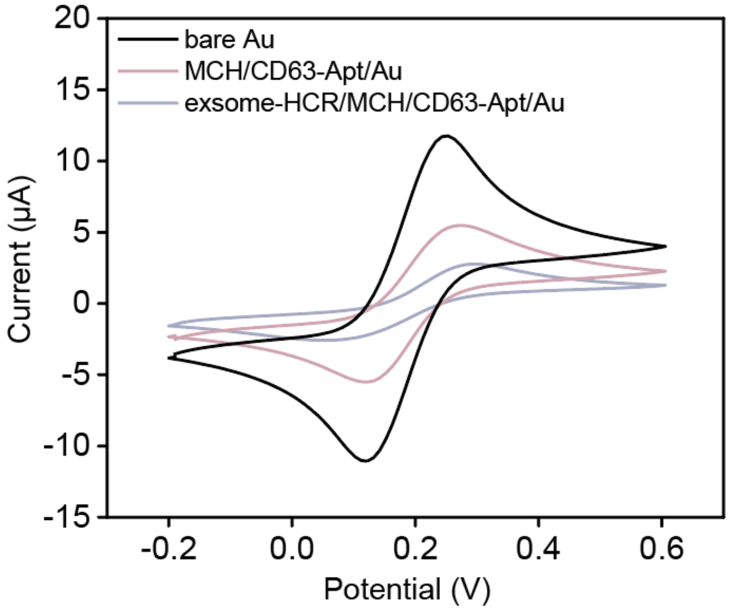

Figure S1. CV curves of bare Au, MCH/CD63-Apt/Au, and exosome–HCR/MCH/CD63-Apt/Au in 0.5 mM $[Fe(CN)_6]^{3-/4}$ solution containing 0.1 M KCl.

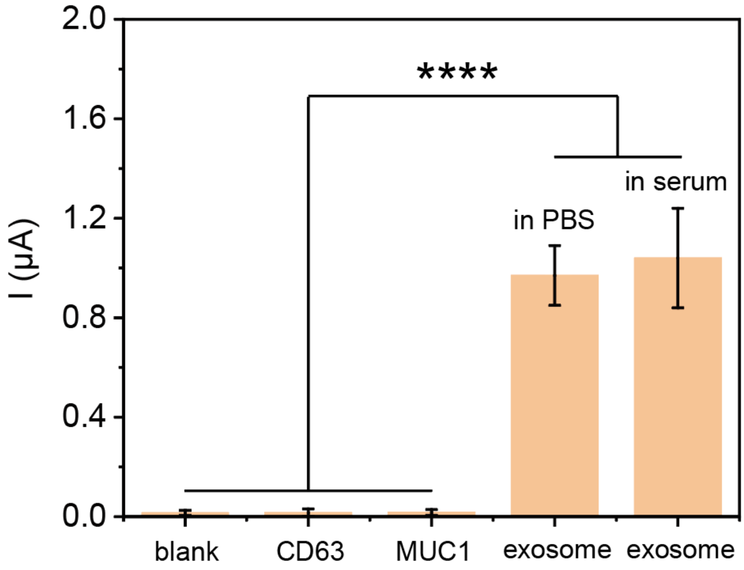

Figure S2. Anti-interference ability of this aptasensor for the detection of exosomes (P≤0.0001).

Table S1. DNA sequences required in the experiment (5' to 3').

Name	Sequences (5' to 3')
CD63-Apt	CACCCCACCTCGCTCCCGTGACACTAATGCTA-SH

MUC1-Apt	GCAGTTGATCCTTTGGATACCCTGGTTTTTTTTTTT GCCGTCGTGCCTTAT
cholesterol probe	AATTCAGCACAATGGATAAGCTACTATAACATA
	AGGCACGACGGCTTTTTTTTTTTGACCCTAAGCATACATGCTCACTGA–cholesterol
HP1	Biotin–ACA ATG GAT AAG CTA CTA TAA CTC TGA AAC GTT ATA GTA GCT TAT CCA TTG TGC TGAATT
HP2	GTT TCA GAG TTA TAG TAG CTT ATC CAT TGT AT GTC TCA CAA TGG ATA AGC TAC TAT AAC–biotin
